# Peripheral T Cell Cytokine Responses for Diagnosis of Active Tuberculosis

**DOI:** 10.1371/journal.pone.0035290

**Published:** 2012-04-16

**Authors:** Johannes Nemeth, Heide-Maria Winkler, Ralph H. Zwick, Catharina Müller, Rudolf Rumetshofer, Lucas Boeck, Otto C. Burghuber, Stefan Winkler

**Affiliations:** 1 Department of Internal Medicine I, Division of Infectious Diseases and Tropical Medicine, Medical University of Vienna, Vienna, Austria; 2 Division of Infectious Diseases and Hospital Epidemiology, University Hospital Zurich, Zurich, Switzerland; 3 Department of Respiratory and Critical Care Medicine, Otto-Wagner-Hospital, Vienna, Austria; 4 Clinic of Pulmonary Medicine and Respiratory Cell Research, University Hospital Basel, Basel, Switzerland; University of Delhi, India

## Abstract

**Background:**

A test for diagnosis of active Tuberculosis (TB) from peripheral blood could tremendously improve clinical management of patients.

**Methods:**

Of 178 prospectively enrolled patients with possible TB, 60 patients were diagnosed with pulmonary and 27 patients with extrapulmonary TB. The frequencies of *Mycobacterium tuberculosis* (MTB) specific CD4^+^ T cells and CD8^+^ T cells producing cytokines were assessed using overnight stimulation with purified protein derivate (PPD) or early secretory antigenic target (ESAT)-6, respectively.

**Results:**

Among patients with active TB, an increased type 1 cytokine profile consisting of mainly CD4^+^ T cell derived interferon (IFN)-γ was detectable. Despite contributing to the cytokine profile as a whole, the independent diagnostic performance of one cytokine producing T cells as well as polyfunctional T cells was poor. IFN-γ/Interleukin(IL)-2 cytokine ratios discriminated best between active TB and other diseases.

**Conclusion:**

T cells producing one cytokine and polyfunctional T cells have a limited role in diagnosis of active TB. The significant shift from a “memory type” to an “effector type” cytokine profile may be useful for further development of a rapid immune-diagnostic tool for active TB.

## Introduction

Tuberculosis (TB) remains to be a global health care problem and together with malaria and HIV is considered to be one of the three key infectious diseases worldwide [1]. The improvement of clinical management of active TB relies primarily on the unambiguous diagnosis of the disease. However, a rapid and straightforward test to confirm or rule out active TB is lacking in clinical routine [2]. Indeed, a diagnostic test for diagnosis of active TB from an easily accessible compartment such as peripheral blood could significantly improve patient management.

Such a test appeared to be in reach after the discovery of *Mycobacterium tuberculosis* (MTB) specific antigens and their use for T cell stimulation assays based on Enzyme Linked Immuno Spot Technique (ELISPOT) and Enzyme-Linked Immunosorbent Assay (ELISA) techniques [3], [4]. Both tests rely on in vitro produced interferon (IFN)- γ as read out and have been shown to introduce increased sensitivity and specificity for the diagnosis of latent TB infection [5]. Disappointingly, however, the tests are not suitable for the diagnosis of active TB [4].

A flow cytometry based read out has been suggested to possibly improve the diagnostic accuracy of MTB specific stimulation assays, because MTB specific T cell subsets producing different types of cytokines can be analysed on a single cell basis [6]. T cells producing single cytokines [7], two cytokines – “polyfunctional T cells [8]” - and three cytokines - “multifunctional T cells [9], [10]” – have been linked to bacterial load and disease activity. More recently CD4^+^ T cells producing single tumor necrosis factor (TNF)-α have been suggested to differentiate between active TB and latent infection [7]. Moreover, cytokines generally regarded as pro-inflammatory such as TNF-α, IFN-γ and Interleukin (IL)-2 were associated with active TB as well as regulatory cytokines like IL-10 and transforming growth factor (TGF)-β [11]–[13]. Thus, we hypothesised that a distinct cytokine profile could be useful for the diagnosis of active TB.

The recent investigation was conducted to prospectively assess sensitivity and specificity of MTB specific, one cytokine producing and polyfunctional T cells in patients with the clinical suspicion of active TB to possibly discover an MTB specific cytokine signature.

## Materials and Methods

### 1. Patients

Patient recruitment was confined to the wards of the Medical University of Vienna (Division of Infectious Diseases and Tropical Medicine) and the Department of Respiratory and Critical Care Medicine at the Otto-Wagner Hospital in Vienna, Austria.

Written informed consent was obtained from all participating individuals. Human experimentation guidelines of the Medical University of Vienna were followed during the clinical research. Ethical clearance was given by the Ethics Committee of the Medical University of Vienna and the Viennese Krankenanstaltenverbund.

Patients presenting with signs and symptoms suggestive of TB were eligible for this study. Human immunodeficiency (HI)-virus infected patients were not included in the study. All study participants had a history of BCG vaccination (in Austria BCG vaccination was stopped in 1990; countries of origin of all other study participants (e.g. Russian Federation, Serbia, Romania, Ukraine, Bulgaria,…) are still administering BCG vaccines. No history of previous TB was reported by any of the patients. The presence of latent MTB-infection was not generally looked at, as it was the purpose of the study to potentially differentiate active TB from other diseases. According to the attending physicians, the pre-test probability for TB in this study population was approximately 30%, taking in account the history, social background, signs and symptoms. Approximately 27 ml of blood were drawn for the isolation of peripheral blood mononuclear cells (PBMC) during initial assessment.

Pulmonary TB was defined by the presence of the following criteria: detection of MTB by culture or PCR in sputum or bronchoalveolar fluid obtained by bronchoalveolar lavage (BAL) and the clinical diagnosis of pulmonary TB with the concomitant initiation of a tuberculostatic drug therapy. Extra-pulmonary TB was defined by the detection of MTB in other tissue than the lungs, the clinical diagnosis of active TB disease and the initiation of treatment.

If MTB was not detectable by culture or PCR, the unambiguous clinical diagnosis and the initiation of a tuberculostatic drug therapy was required to fulfil the diagnosis of active TB. In these cases, active TB infection was diagnosed by the presence of necrotizing granulomatous inflammation without other causes, clinical history suggestive of active TB, including at least three of the following symptoms: night sweat, unintended weight loss, malaise, fever, lassitude and known exposure to open TB. Additionally, the absence of another diagnosis and the clinical response to tuberculostatic drugs was evaluated.

Importantly, doctors involved in clinical management and decision making were completely unaware of the results of the stimulation assays.

### 2. Methods

**Figure 1:**
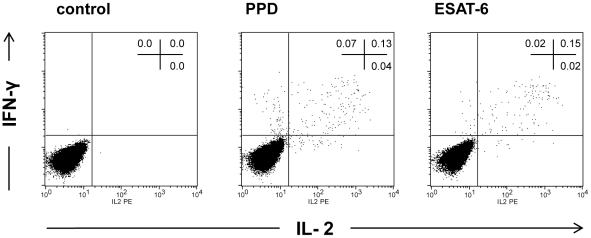
Representative dot plots. Representative two-parameter dot plots of a patient with extrapulmonary TB (urogenital TB) indicating the frequency of PPD and ESAT-6-specific CD4^+^ T cells expressing IFN-γ and/or IL-2, respectively. PBMC were incubated with medium alone (control), PPD and ESAT-6, respectively.

PBMC were isolated from heparinized blood by ficoll-diatrizoate centrifugation, and plated out into 24-well plates (BD Falcon, Mountain View, CA, USA) at 2×10^6^ per well. Cells were cultured in ultra-culture medium (UCM) (Bio Whittaker, Walkersville, MD, USA) supplemented with L-glutamine (2 mM/L; Sigma, St. Louis, MI, USA), gentamicin (170 mg/l; Sigma) and 2-mercaptoethanol (3.5 µl/L; Merck, Darmstadt, Germany) for 18 h at 37°C in 5% CO2 and stimulated with purified protein derivate (PPD) (Statens Serum Institute, Copenhagen, Denmark), at a final concentration of 10 µg/mL or with early secretory antigenic target (ESAT)-6 (Statens Serum Institute, Copenhagen, Denmark) with a final concentration of 5 µg/ml. In order to amplify TCR signalling and to facilitate the initial phase of the T-cell activation, the co-stimulatory MAb CD28 (Pharmingen San Diego, CA, USA), was added at a final concentration of 5 µg/mL. Brefeldin A (10 µg/mL final concentration, Sigma) was added after 6 h to block protein secretion. After 18 h, cells were harvested on ice, washed twice in phosphate-buffered saline (PBS), and fixed with 2% formaldehyde (1 mL per 2×10^6^ cells) for 20 minutes. After two additional washes in PBS, the cells were re-suspended in Hank's balanced salt solution (supplemented with 0.3% bovine serum albumin and 0.1% sodium-azide). The cells were washed twice with PBS and made permeable with saponin (0.1%; Sigma), re-suspended with 50 µL of saponin-buffered diluted antibodies and incubated for 25 minutes in the dark. The following monoclonal antibodies were used: Anti-CD4, PerCP and APC labelled, Anti-CD8, PerCP labelled (Becton and Dickinson, Mountain View, CA); Anti-TGF-β, PE labelled (IQProducts, Groningen, The Netherlands); MAb IFN-γ (clone: B 27), fluorescein–isothiocyanate (FITC) labelled; MAb IL–2 (clone: MQ1-17H12), PE labelled; MAb IL-10 (clone: JES3-9D7), PE labelled; MAb TNF–α (clone: MAB -11), PE labelled (all Becton and Dickinson). Four-color staining was performed, and a minimum of 10^5^ PBMCs were analysed on a FACS-Calibur (Becton Dickinson) equipped with a two-laser system (488- and 630-nm wavelength, respectively) (Figure 1). All cytokine combinations were stained in conjunction with CD4 and CD8. The data were analysed with CELL-Quest software (Becton Dickinson) and the results were expressed as the percentage of cytokine-producing cells in each CD4^+^ or CD8^+^ population. To assure specificity, spontaneous cytokine production in control wells was subtracted from cytokine production after stimulation with PPD or ESAT-6.

### 3. Statistics

Statistical analysis was performed using SPSS 15.0 for Windows, SPSS Inc., Chicago. The independent-samples T-test was applied to screen for differences between 2 groups. A one-way between-groups analysis of variance (ANOVA) with a Tukey post-hoc test was used for 3 groups. Direct logistic regression was calculated between the following groups: pulmonary TB and non-TB diseases, extrapulmonary TB and non-TB diseases, pulmonary and extrapulmonary TB. Cytokine ratios were calculated by dividing the total percentages of the respective cytokine. Receiver-operating-characteristic curves (ROC) were calculated and expressed as areas under the curve (AUC), with an asymptotic 95% confidence interval (CI). A p-value of <0.05 (two tailed) was considered significant.

## Results

### 1. Patients

A total of 178 patients were included in the study. 112 were men (62.9%) and 66 were women (37.1%). 60 patients were classified as suffering from pulmonary TB, 27 from extrapulmonary TB and 91 from non-TB diseases. Patient details are depicted in Table 1. Clinical characteristics and total MTB specific CD4^+^IFN-γ^+^ T cells from peripheral blood of 25 patients were published already [14].

**Table 1:** Patient characteristics.

diagnosis		number of individuals, total: 178	male/female total: 112/66	age (median, min–max) total: 47.4 (14.8–86.5)	MTB confirmation total: 85.1%	histological evidence of TB
pulmonary TB		60	39/21	42.8 (17.8–86.5)	55 (91.7%)	11 (18.3%)
extra pulmonary TB (n = 27)	bone TB	2	1/1	24.1 (21.3–26.9)	1 (100%)	
	TB lymphadenopathy	3	1/1	29.9 (25.8–33.5)	3 (100%)	1 (50%)
	miliary TB	2	0/2	31.8 (24.7–38.9)	2 (100%)	
	peritoneal TB	4	3/1	31.7 (14.7–84.2)	1 (25%)	3 (75%)
	soft tissue TB	5	2/3	58.8 (29.9–82.6)	4 (80%)	3 (60%)
	liver TB	1	0/1	72	1 (100%)	
	TB meningitis	2	1/1	38 (18.2–57.8)	2 (100%)	
	joint TB	2	1/1	57.8 (46.1–69.5)	1 (50%)	2 (100%)
	TB pericarditis	1	0/1	47.3	1 (100%)	1 (100%)
	TB pleuritis	3	2/1	31.3 (17.8–52)	1 (33.3%)	
	urogenital TB	2	0/2	44.8 (22.6–67)	1 (100%)	1 (100%)
non-TB diseases (n = 91)	bacterial peritonitis	1	1/0	53.4		
	cancer	26	17/9	58.4 (30.9–84.0)		
	bronchitis	2	1/1	39.4 (36.8–42.1)		
	CKR changes of unknown origin	13	10/3	53 (28–64.4)		
	pneumonia	26	18/8	46.3 (20.6–78.1)		
	liver cirrhosis	1	1/0	56.2		
	COPD^1^	4	3/1	51.8 (50–69.8)		
	viral encephalitis	1	1/0	56.4		
	fibrosis of the lung	3	1/3	26.0 (24.7–59.8)		
	*M.chelonae* 2	1	0/1	73.2		
	*M.kansasii* 2	2	0/2	47.7 (47.6–47.9)		
	*M.xenopi* 2	2	1/1	64.7 (51.1–78.5)		
	idiopathic pericarditis	1	0/1	37.8		
	pneumoconiosis	1	1/0	77.2		
	rheumatoid arthritis	1	1/0	42.8		
	sarcoidosis	2	0/2	68.7 (66.8–70.6)		
	septic arthritis	1	1/0	53.2		
	idiopathic polyserositis	2	1/1	53 (24.3–81.7)		
	silicosis	1	0/1	42.7		

1 chronic obstructive pulmonary disease.

2 infection with non-tuberculous mycobacteria.

### 2. Frequencies of MTB-specific T cells

#### 2.1. Differences between pulmonary TB and diseases other than TB

**Figure 2:**
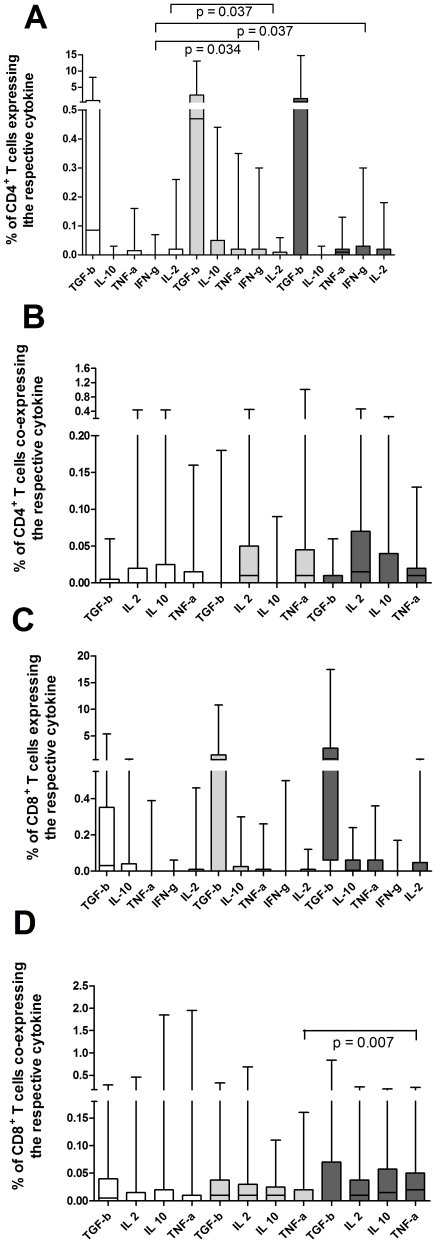
Frequencies of ESAT-6 specific T cells. Frequencies of single cytokine producing T cells and multifunctional T cells of 60 patients with pulmonary TB (light grey), 27 extrapulmonary TB (dark grey) and 91 with non-tuberculous diseases (white) after overnight stimulation with ESAT-6 are depicted. Boxes and whiskers are shown; the black line marks the median. Differences between patient groups are marked with a bar. A significantly increased frequency of CD4^+^ T cells expressing IFN-γ (0; 0–0; 0–0.07 vs. 0; 0–0.02; 0–0.3 or 0; 0–0.03; 0–0.3 [median; 25%–75% percentile; min–max], respectively. p = 0.034) and a decreased frequency of CD4^+^ T cells expressing IL-2 (0; 0–0.02; 0–0.26 vs. 0; 0–0.01; 0–0.06 [median; 25%–75% percentile; min–max]. p = 0.037) were found in patients with pulmonary TB when compared to diseases other then TB. CD8^+^ T cells expressing IFN-γ and TNF-α^+^ (0; 0–0.02; 0–0.16 vs. 0.02 ; 0–0.05; 0–0.23 [median; 25%–75% percentile; min–max]. p = 0.041) were different when pulmonary and extrapulmonary TB were compared. An independent-samples t-test was used to test for significance.

**Figure 3:**
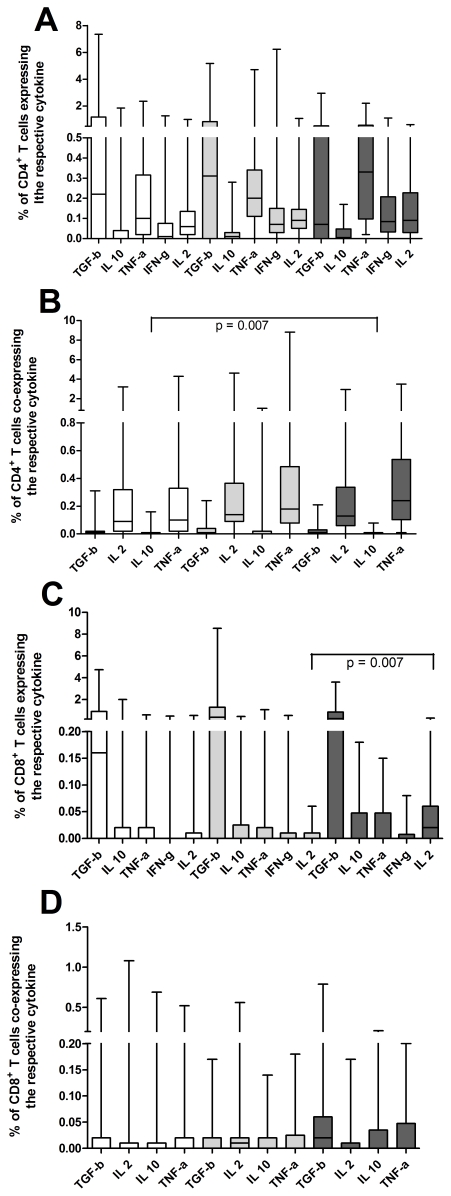
Frequencies of PPD specific T cells. Frequencies of single cytokine producing T cells and multifunctional T cells of 60 patients with pulmonary TB (light grey), 27 extrapulmonary TB (dark grey) and 91 with non-tuberculous diseases (white) after overnight stimulation with PPD are depicted. Boxes and whiskers are shown; the black line marks the median. CD4^+^IFN-γ^+^IL-10^+^ T cells (0.01 ; 0.02–0.33; 0–0.16 vs. 0; 0–0.01; 0–0.08 [median; 25%–75% percentile; min–max]. p = 0.007) were significantly different when extrapulmonary TB was compared to other diseases then TB. Comparing pulmonary and extrapulmonary TB, PPD specific CD8^+^ T cells expressing IL-2 (0.14 ; 0.09–0.365; 0–4.61 vs. 0.13; 0.06–0.33; 0–2.93 [median; 25%–75% percentile; min–max]. p = 0.011) were significantly different. Differences between patient groups are marked with a bar. An independent-samples t-test was used to test for significance.

An independent-samples t-test showed an increased frequency of CD4^+^ T cells expressing IFN-γ (p = 0.034) and a significantly decreased frequency of CD4^+^ T cells expressing IL-2 (p = 0.037) - both ESAT-6 specific - in patients with pulmonary TB when compared to diseases other then TB (Figure 2). With regard to PPD stimulated T cells no differences were found between TB and non-TB patients (Figure 3).

**Figure 4:**
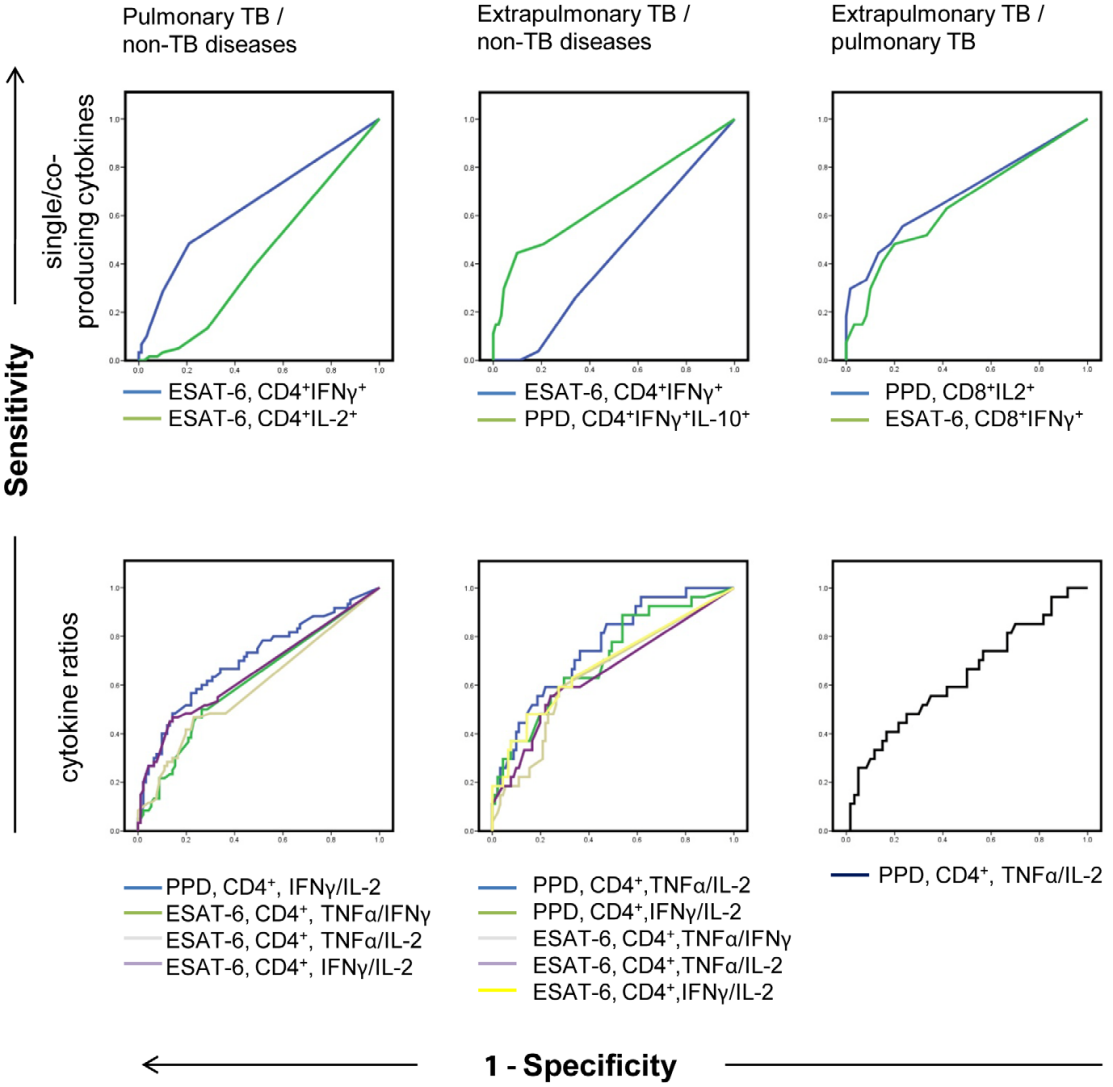
ROC curves. Receiver-operating-characteristic curves (ROC) were calculated for every significantly different cytokine and cytokine ratio. AUCs are summarized in Table 2.

Direct logistic regression was performed to assess the impact of each single factor on the likelihood that patients would have pulmonary TB or not. The full model containing age, sex, CD4^+^IFN-γ^+^ T cells, CD4^+^IL-2^+^ T cells was statistically significant, X^2^ (4 df) = 34.465, p<0.001. The model as a whole correctly classified 72.7% of the cases. Age (p<0.001), CD4^+^IFN-γ^+^ T cells (p = 0.033), CD4^+^IL-2^+^ T cells (p = 0.032) made a unique statistically significant contribution to the model. A Receiver-operating-characteristic curve (ROC) was calculated and expressed as area under the curve (Figure 4), with an asymptotic 95% confidence interval (CI).

#### 2.2. Differences between extrapulmonary TB and diseases other than TB

An independent-samples t-test showed decreased frequency of PPD specific CD4^+^IFN-γ^+^IL-10^+^ T cells (p = 0.007) and an increased frequency of ESAT-6 specific CD4^+^IFN-γ^+^ T cells (p = 0.037) (Figures 2, 3). Direct logistic regression was performed to assess the impact of each single factor on the likelihood that patients would have extrapulmonary TB or not. The full model containing age, sex, PPD specific CD4^+^IFN-γ^+^IL-10^+^ T cells and ESAT-6 specific CD4^+^IFN-γ^+^ T cells was statistically significant, X^2^ (7 df) = 33.2 p<0.001. The model as a whole correctly classified 82.2% of the cases. Age (p = 0.006) and CD4^+^IFN-γ^+^ T cells (p = 0.015) made a unique statistically significant contribution to the model. A Receiver-operating-characteristic curve (ROC) was calculated and expressed as area under the curve (Figure 4), with an asymptotic 95% confidence interval (CI) (summarized in Table 2).

**Table 2:** Summary of receiver-operating-characteristic curves (ROC).

			Antigen	AUC	S.E.	Sig.	95% CI	
pulmonary TB/other diseases cytokines	CD4^+^	IFN-γ^+^	ESAT-6	0.644	0.047	0.003	0.551	0.736
		IL-2^+^		0.428	0.047	0.136	0.337	0.519
pulmonary TB/other diseases cytokine-ratios	CD4^+^	IFN-γ/IL-2	PPD	0.704	0.045	<0.001	0.617	0.792
		TNF-α/IFN-γ	ESAT-6	0.613	0.048	0.019	0.519	0.706
		TNF-α/IL-2		0.593	0.049	0.054	0.497	0.688
		IFN-γ/IL-2		0.654	0.048	= 0.001	0.560	0.747
extrapulmonary TB/other diseases cytokines	CD4^+^	IFN-γ^+^IL-10^+^	PPD	0.439	0.059	0.349	0.324	0.555
		IFN-γ^+^	ESAT-6	0.665	0.067	0.009	0.534	0.795
extrapulmonary TB/other diseases cytokine-ratios	CD4^+^	TNF-α/IL-2	PPD	0.756	0.051	<0.001	0.657	0.856
		IFN-γ/IL-2		0.712	0.057	0.001	0.600	0.824
		TNF-α/IFN-γ	ESAT-6	0.648	0.062	0.020	0.527	0.768
		TNF-α/IL-2		0.652	0.064	0.017	0.525	0.778
		IFN-γ/IL-2		0.685	0.063	0.004	0.561	0.809
extrapulmonary TB/pulmonary TB cytokines	CD8^+^	IL-2^+^	PPD	0.689	0.067	0.005	0.558	0.819
	CD8^+^	IFN-γ^+^TNF-α^+^	ESAT-6	0.645	0.067	0.031	0.514	0.776
extrapulmonary TB/pulmonary TB cytokine-ratios	CD4^+^	TNF-α/IL-2	PPD	0.634	0.066	0.045	0.505	0.765

Legends: AUC: area under the curve; S.E.: standard error; Sig.: significance level; CI: confidence interval.

#### 2.3. Differences between extrapulmonary and pulmonary TB

An independent-samples t-test showed an increased frequency of PPD specific CD8^+^ T cells expressing IL-2 (p = 0.011) and ESAT-6 specific CD8^+^ T cells expressing IFN-γ and TNF-α^+^ (p = 0.041) (Figures 2, 3). Direct logistic regression was performed to assess the impact of each single factor on the likelihood that patients would have extrapulmonary or pulmonary TB. The full model containing age, sex, PPD specific CD8^+^IL-2^+^ T cells and ESAT-6 specific CD8^+^IFN-γ^+^TNF-α^+^ T cells was statistically significant, X^2^ (4 df) = 22.286, p<0.001. The model as a whole correctly classified 74.4% of the cases. PPD specific CD8^+^IL-2^+^ T cells (p = 0.004) and ESAT-6 specific CD8^+^IFN-γ^+^TNF-α^+^ T cells (p = 0.031) made a unique statistically significant contribution to the model. A Receiver-operating-characteristic curve (ROC) was calculated and expressed as area under the curve (Figure 4), with an asymptotic 95% confidence interval (CI) (summarized in Table 2).

#### 2.4. Differences of cytokine ratios between groups

**Figure 5:**
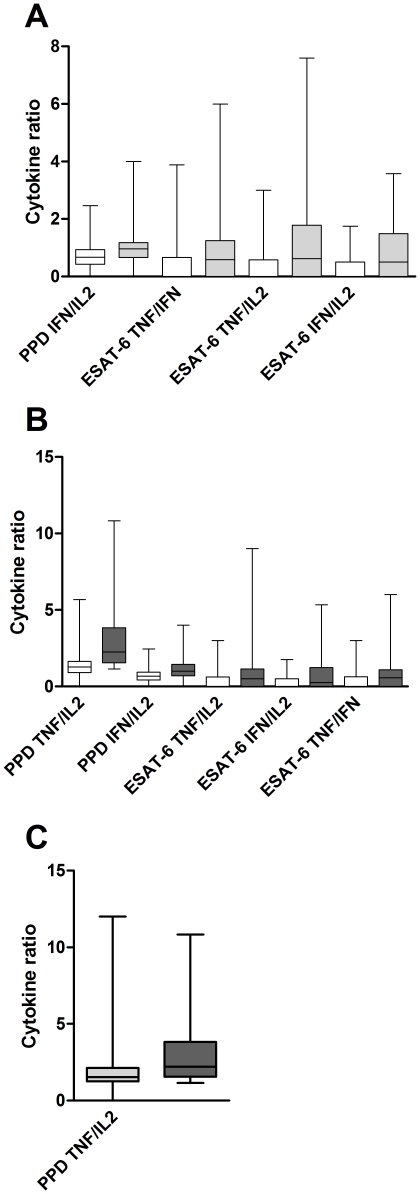
Cytokine ratios. Significant differences between different cytokine ratios are depicted, detected with a one way between-groups analysis of variance using the tukey test for post hoc analysis. Data from 60 patients with pulmonary TB (light grey), 27 extrapulmonary TB (dark grey) and 91 with non-tuberculous diseases (white) are depicted. Boxes and whiskers are displayed, the black line marks the median. Percentile ranges are additionally shown in Tables 3, 4, 5. (A) shows statistically significant differences between non-tuberculous diseases and pulmonary TB: PPD induced IFN-γ/IL-2 (p<0.001), ESAT-6 induced TNF-α/IFN-γ (p = 0.048), TNF-α/IL-2 (p = 0.03), IFN-γ/IL-2 (p = 0.005), all CD4^+^ T cell derived. (B) shows statistically significant differences between non-tuberculous diseases and extrapulmonary TB: PPD induced TNF-α/IL-2 (p<0.001), IFN-γ/IL-2 (p = 0.001), ESAT-6 induced TNF-α/IFN-γ (p = 0.026), TNF-α/IL-2 (p = 0.008), IFN-γ/IL-2 (p<0.001), CD4^+^ T cell derived. (C) shows statistically significant differences between pulmonary TB and extrapulmonary TB: PPD induced TNF-α/IL-2 (p = 0.001), CD4^+^ T cell derived.

A one way between-groups analysis of variance using the tukey test for post hoc analysis was conducted to explore the impact of different cytokine ratios between pulmonary TB, extrapulmonary TB and other diseases than TB (displayed in Figure 5, the percentile ranges of the box and whiskers are depicted in Tables 3, 4, 5).

**Table 3:** Cytokine ratios pulmonary TB/non-tuberculous diseases.

	PPD IFN-γ/IL-2		ESAT-6 TNF-α/IFN-γ		ESAT-6 TNF-α/IL-2		ESAT-6 IFN-γ/IL-2	
	non-TB	pul-TB	non-TB	pul-TB	non-TB	Pul-TB	non-TB	pul-TB
Minimum	0.0	0.0	0.0	0.0	0.0	0.0	0.0	0.0
25% Percentile	0.4325	0.6650	0.0	0.0	0.0	0.0	0.0	0.0
Median	0.6700	0.9600	0.0	0.5850	0.0	0.6250	0.0	0.5000
75% Percentile	0.9300	1.183	0.6600	1.250	0.5750	1.785	0.5000	1.500
Maximum	2.460	4.000	3.880	6.000	3.000	7.600	1.750	3.580

**Table 4:** Cytokine ratios extrapulmonary TB/non-tuberculous diseases.

	PPD TNF-α/IL-2		PPD IFN-γ/IL2		ESAT-6 TNF-α/IL-2		ESAT-6 IFN-γ/IL-2		ESAT-6 TNF-α/IFN-γ	
	non-TB	extra-TB	non-TB	extra-TB	non-TB	Extra-TB	non-TB	extra-TB	non-TB	extra-TB
Minimum	0.0	1.140	0.0	0.0	0.0	0.0	0.0	0.0	0.0	0.0
25% Percentile	0.9050	1.540	0.4350	0.7075	0.0	0.0	0.0	0.0	0.0	0.0
Median	1.270	2.270	0.6700	1.000	0.0	0.5000	0.0	0.2500	0.0	0.5714
75% Percentile	1.640	3.840	0.9350	1.438	0.6150	1.150	0.5000	1.230	0.6250	1.091
Maximum	5.670	10.83	2.460	4.000	3.000	9.000	1.750	5.330	3.000	6.000

**Table 5:** Cytokine ratios pulmonary TB/extrapulmonary TB.

	PPD TNF-α/IL-2	
	pul-TB	extra-TB
Minimum	0.0	1.140
25% Percentile	1.243	1.540
Median	1.525	2.200
75% Percentile	2.133	3.818
Maximum	12.00	10.83

The following cytokine ratios were found to be different between non-TB diseases and pulmonary TB: PPD induced IFN-γ/IL-2 (p<0.001) and ESAT-6 induced TNF-α/IFN-γ (p = 0.048). TNF-α/IL-2 (p = 0.03), and IFN-γ/IL-2 (p = 0.005). All were CD4^+^ T cell derived.

The following cytokine ratios were found to be different between non-TB diseases and extrapulmonary TB: PPD induced TNF-α/IL-2 (p<0.001), IFN-γ/IL-2 (p = 0.001) and ESAT-6 induced TNF-α/IFN-γ (p = 0.026), TNF-α/IL-2 (p = 0.008), and IFN-γ/IL-2 (p<0.001). All were CD4^+^ T cell derived.

The following cytokine ratio was found to be different between pulmonary and extrapulmonary TB: PPD induced TNF-α/IL-2 (p = 0.001), CD4^+^ T cell derived.

For each cytokine ratio, a receiver-operating-characteristic curve (ROC) was calculated and expressed as area under the curve with an asymptotic 95% confidence interval (CI). The highest AUCs to discriminate between pulmonary TB and other diseases −0.704 and 0.654 - were the ratios of IFN-γ divided by IL-2, induced by PPD and ESAT-6, respectively. The highest AUCs to discriminate between extrapulmonary TB and other diseases −0.756 and 0.712 - were the ratios of PPD induced IFN-γ and TNF-α, divided by IL-2, respectively (Figure 4, summarized in Table 2).

## Discussion

In this prospective clinical study a type 1 cytokine profile specific for both pulmonary and extrapulmonary TB was detected, consisting of a robust production of IFN-γ by MTB-specific CD4^+^ T cells. Neither one cytokine producing T cells nor polyfunctional T cells appeared to have a useful diagnostic value on their own. In contrast, a relative shift from IL-2 towards IFN-γ production in T cells was associated with active TB, suggesting that cytokine ratios might introduce more discriminatory power than assessing single cytokine producing T cells.

Differences between pulmonary TB and other diseases were restricted to increased frequencies of CD4^+^IFN-γ^+^ T cells and decreased frequencies of CD4^+^IL-2^+^ T cells, contributing independently to the logistic regression model. Disappointingly, the AUC for the respective cytokines after ROC analysis were comparatively low, limiting thereby their diagnostic value. The independent increase of CD4^+^IFN-γ^+^ “effector” T cells during active – both pulmonary and extrapulmonary – TB is backed by the recently published transcriptional signature for active TB, consisting mainly of an interferon gene profile [15].

The decrease of IL-2 producing T cells – a functional “memory” equivalent [16] - is in line with published data, showing that patients with active TB had decreased frequencies of single IL-2 producing T cells if compared to their house hold contacts [17].

In contrast to CD4^+^IFN-γ^+^ T cells, TNF-α producing T cells were not independently associated with active TB. In fact, TNF-α producing CD4^+^ T cells were not even statistically different between patient groups, questioning their value as a diagnostic tool. These results differ significantly from previously published results, showing high association between active TB and T cells producing single TNF-α [7], [12]. This discrepancy could be due to two main differences.

Firstly and in contrast to these previous investigations, the control group in our recent study consisted of patients who suffered from other diseases than TB. The control groups in the above mentioned studies were latently infected but apparently healthy individuals. Thus, it is intriguing, that the value of single TNF-α producing T cells for diagnosis of active TB is questionable in different patient groups with initial suspicion of active TB.

Secondly and in contrast to previous investigations, T cells were co-stained with IFN-γ only and not with any other cytokines [7], [9], [18]. Therefore, we are not able to directly compare our results with the data published in the literature as “single” cytokine producing T cells. Consequently, we used the term “one cytokine producing T cell” as opposed to “single cytokine producing T cell”. Nevertheless, the complete lack of difference between patient groups - as shown for example for TNF-α - questions the actual usefulness of individual cytokines in general despite minor differences in read out.

The later administration of Brefeldin A (6 hours in our study instead of 1–2 hours, as reported in [7]) to the stimulatory assay does not explain the lack of discrimination between the subject groups, as we have found reliable pro- as well as anti-inflammatory responses after PPD and ESAT-6 stimulation in all groups. Additional experiments have also not shown substantial differences in cytokine expression when Brefeldin A was added 4 hours earlier (data not shown).

The only significantly increased multifunctional T cell subpopulations were CD4^+^IFN-γ^+^IL-10^+^ T cells, when TB patients were compared to patients with non-TB diseases. Given their relative reduction in patients with extrapulmonary TB, it is tempting to speculate about immune-regulatory properties of this cellular subpopulation. However, CD4^+^IFN-γ^+^IL-10^+^ T cells did not contribute independently to the logistic regression model, questioning their relevance for diagnostic purposes.

To date, the available data on multi- and polyfunctional MTB specific T cells during active TB are inconsistent. Different reports suggested increased [17], decreased [9] and no differences [10] of multi-functional T cells in active TB if compared to different control groups. In our clinical cohort, including the by far largest number of patients published to date, multifunctional T cells were not associated with the active TB. This finding could be either explained by the decrease of multiple cytokine producing T cells in the TB patient group caused by the exhaustion of T cells during active TB, as suggested earlier in analogy to viral infections [19], [20]. On the contrary, however, this result may also be explained by an increase of multifunctional T cells in the patient group suffering from non-TB diseases. Indeed, polyfunctional T cells were associated with chronic viral infections and vaccine memory [10], [21]–[23].

Differences between extra-pulmonary and pulmonary TB were minor when assessing one cytokine expressing CD4^+^ T cells and – unexpectedly - restricted to differences among CD8^+^ T cells. These differences have not been described before, possibly because differences between pulmonary and extrapulmonary TB were not subject of many studies. The current data available suggest, for example, that miliary TB is associated with an immune-regulatory phenotype [24], whereas pleural TB is associated with a strong, localized type 1 immune response [4], [12]. Regarding CD8^+^ T cells, different concepts exist on their role during infection with MTB [25]. CD8^+^ T cells produce IFN-γ and TNF-α upon exposure to different MTB specific peptides in latent infected individuals [8] and have been suggested to be associated with disease progression [9].

The distinction between pulmonary TB and extrapulmonary TB might seem arbitrary, given that the latter is quite a heterogeneous group of disease manifestations. This classification, however, has significant clinical impact, because extrapulmonary TB is thought not to be contagious or at least much less so if compared to pulmonary TB [26] and has different treatment periods. The recent data suggest that immunological differences between the two manifestations of MTB associated diseases exist, which have to be considered in both immune based diagnostics as well as vaccine trials.

To our surprise, the highest AUCs after ROC analysis to discriminate pulmonary and extrapulmonary TB from other diseases were PPD induced cytokine ratios of IFN-γ/IL-2 and TNF-α/IL-2, respectively. A higher IFN-γ/IL-2 ratio has been reported previously to be associated with untreated TB [18], [27], [28]. This might be related to a generally higher frequency of PPD specific T cells in peripheral blood as compared to ESAT-6 specific T cells [6], [12]–[14], [29].

In our study cohort, PPD stimulation was followed by a relative increase of IFN-γ over IL-2, which discriminated best between patient groups and confirmed the presence of an IFN-γ secreting, “effector” phenotype of CD4^+^ T cells in patients with active TB [16], [30]. Assessment of the TNF-α/IL-2 ratio derived of CD4^+^ T cells revealed increased TNF-α in patients suffering from extra-pulmonary TB, suggesting a more pro-inflammatory cytokine profile if compared to pulmonary TB. Thus, the use of cytokine ratios rather than single cytokine measurements could help to overcome the pronounced, inter-individual variability of cytokine responses that currently limit their usefulness for immune-diagnosis of active TB.

Our study has some drawbacks. The study was not designed to discriminate between active TB and latent infection but for defining active TB cases. Moreover, the study design did not allow interfering or suggesting any interventions which could alter patient management. Therefore and in accordance with international guidelines, a TST was not routinely administered. As a result, the poor diagnostic performance of MTB specific T cells for the diagnosis of active TB could also be explained by a high frequency of latently infected individuals, who also have MTB specific, polyfunctional T cells detectable in peripheral blood [31]. However, this fact does not alter the conclusions drawn from our study, because active TB was clearly defined in a large number of patients. Whether the T cell response of non-TB patients is caused by latent TB infection, non-tuberculous mycobacteria or by unspecific immunological activation during neoplastic disease is insignificant for a tool aiming at defining active TB out of the large group of TB suspects.

Taken together, our recent study shows in a large clinical cohort that neither pro- nor anti-inflammatory T cell derived cytokines are able to discriminate between TB and non-TB-diseases sufficiently to be suitable as a diagnostic tool. However, cytokine ratios could introduce an improvement in sensitivity and specificity if compared to absolute cytokine amounts, offering new possibilities for immune-diagnosis of active TB.
